# Organ-preserving surgery for male distal primary urethral carcinoma: A case report from a tertiary hospital in Ethiopia

**DOI:** 10.1016/j.ijscr.2025.110983

**Published:** 2025-01-29

**Authors:** Alemu Bedeado Hirpo, Sena Sefara Akasa, Mensur Mohammed Ahmed, Masresha Solomon Dino, Wondweson Alemu Molla, Mintesnot Yitagesu Kidane

**Affiliations:** a Urology Division, Department of Surgery, Saint Paul's Hospital Millennium Medical College, Addis Ababa, Ethiopia; b Department of Pathology, Saint Paul's Hospital Millennium Medical College, Addis Ababa, Ethiopia

**Keywords:** Primary urethral carcinoma, Organ-preserving surgery, Partial glansectomy, Augmented perineal urethrostomy

## Abstract

**Introduction and importance:**

Primary urethral carcinoma (PUC) is a rare cancer, comprising less than 1 % of all genitourinary malignancies, with a male predominance (3:1 ratio), and typically affects those over 75 years of age. PUC shows varied histological subtypes based on their location and sex. The prognosis depends on age, tumor grade, TNM stage, histology, and site. Organ-preserving surgery is for distal disease becoming a preferred option.

**Case presentation:**

We report the surgical management of a male patient presenting with a history of urethral meatal ulcer, bloody urethral discharge, dysuria, and urine spraying, initially misdiagnosed as a persistent herpes ulcer. Examination revealed a 1 × 2 cm erythematous plaque over the urethral meatus. Pelvic magnetic resonance imaging (MRI) showed a 1.7 × 1.7 cm lesion in the distal penile urethra invading the corpus spongiosum, and a biopsy confirmed squamous carcinoma in situ. The patient underwent partial glansectomy and anterior urethrectomy, but pathology showed well-differentiated squamous cell carcinoma with positive margins. The patient was successfully treated with a total anterior urethrectomy, partial glansectomy, and augmented perineal urethrostomy.

**Clinical discussion:**

PUC (primary urethral carcinoma) presents nonspecifically, requiring high suspicion for diagnosis. Historically, treatment included total penectomy with cystoprostatectomy for proximal tumors and partial or radical penectomy for distal tumors. Distal tumors often have better outcomes, and organ preservation surgery is possible for selected patients, with no local recurrence in those treated with additional surgery or adjuvant radiation for positive margins.

**Conclusions:**

PUC is a rare urological malignancy that is challenging to diagnose and treat. Clinical stage and tumor location are critical prognostic factors for urethral carcinoma in men. Organ-preserving surgery is the preferred treatment for distal disease, with a 5-year overall survival rate of approximately 50 %.

## Highlights

• PUC is a rare cancer, and sparse data is available on its pathogenesis and management.
• Nonspecific presentation and initially misdiagnosed with a persistent herpes ulcer.
• Diagnosis requires a high index of suspicion due to nonspecific presentation and rarity of the case.
• The report adds to the limited literature on PUC Penis-preserving surgery, prompting further research.

## 1 Introduction

Primary urethral carcinoma (PUC) is rare, accounting for less than 1 % of all genitourinary malignancies. There is a male predominance of 3:1, with a peak incidence in individuals over 75 years [1,2].

PUCs exhibit a variety of histological subtypes, which vary based on anatomical location and sex. In males, the most common histology is urothelial carcinoma (UC) (54–65 %), followed by squamous cell carcinoma (SCC) (16–22 %), and adenocarcinoma (AC) (10–16 %). In females, the most common subtype is AC (38–46.7 %), followed by SCC (25.4–28 %), UC (24.9–28 %), and other histological entities (6 %) [[1], [2], [3], [4]].

In males, the histological subtype of PUC varies according to the site of origin. Most posterior urethral cancers are urothelial carcinomas, whereas more than 80 % of anterior urethral cancers are squamous cell carcinomas. Among the anterior urethral tumors, 60 % are located in the bulbar urethra and 30 % in the pendulous urethra, with adenocarcinoma being more common in the bulbar urethra [1,3,5].

The risk factors for PUC are multifactorial and differ slightly between men and women. In men, chronic inflammation from a history of sexually transmitted infections (specifically HPV-16), urethral stricture, irritation from intermittent catheterization, and a history of radiation therapy have all been shown to increase the risk of PUC [6,7]. In women, sexually transmitted infections, chronic inflammation, and irritation due to tract infections are risk factors. Additionally, urethral diverticula are associated with an increased risk of developing female PUC, especially adenocarcinoma [1].

Age, grade, TNM stage, histology, and tumor site have been suggested as predictors of cancer-specific survival (CSS), with tumor stage and site being the predominant prognostic factors. Higher-stage tumors have a worse prognosis, as do those arising in the posterior urethra compared to the anterior urethra. Among the histological types, adenocarcinoma is considered the most aggressive because of its high lymph node metastasis rate, followed by squamous cell carcinoma and urothelial carcinoma [1,4,7,8].

Historically, primary urethral carcinoma has been managed by partial or radical penectomy for distal tumors or total penectomy with cystoprostatectomy for proximal tumors [9]. However, Organ-preserving surgical procedures in patients with distal primary urethral cancer aim to achieve an optimal balance between oncological efficacy and maintenance of disease control, while preserving urinary and sexual function, thereby enhancing the patient's overall quality of life. This work has been reported following SCARE criteria [10].

## 2 Case report

A 67-year-old male patient presented to our urology clinic with a complaint of urethral meatal ulcer, bloody urethral discharge, dysuria, and urine spraying for 3 months, after being treated as persistent herpes ulcer for 2 months with acyclovir 400 mg 5×/day, foban ointment by Dermatologist but no improvement. He was claimed to have multiple sexual partners and a history of treatment for sexually transmitted diseases.

**Fig. 1:**
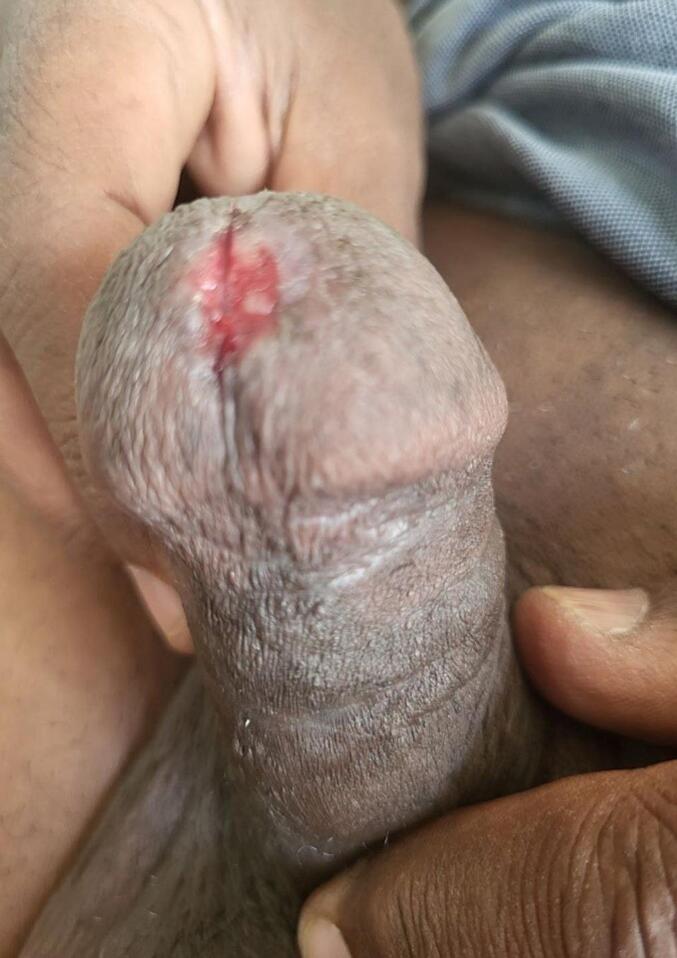
Erythematous plaque over the urethral meatus.

**Fig. 2:**
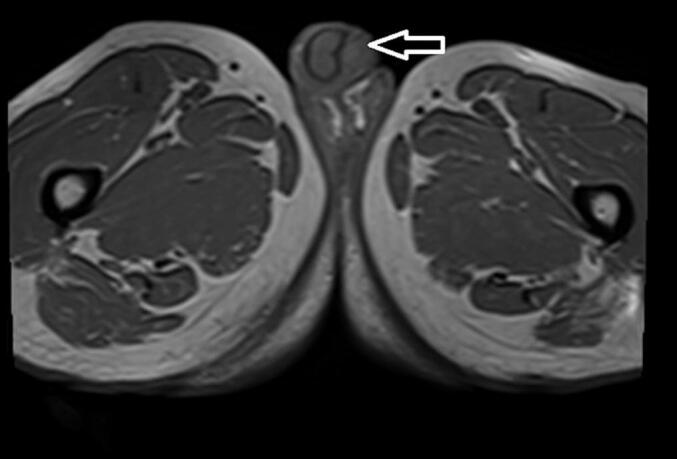
Axial pelvic MRI arrow head showing T1 intermediate intense round lesion at distal penile invading corpus spongiosum.

**Fig. 3:**
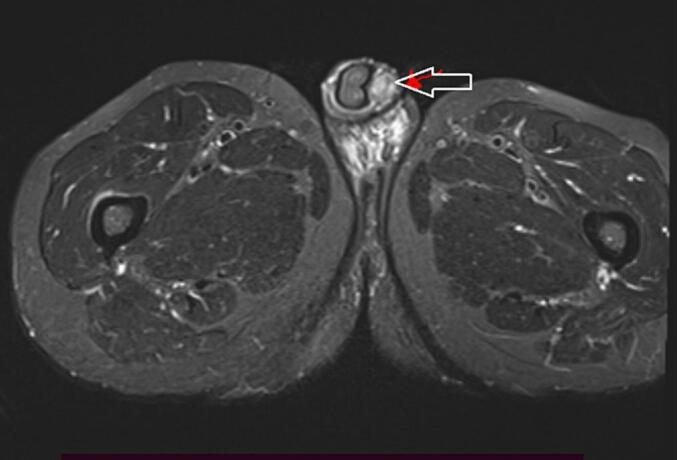
Axial pelvic MRI arrow head showing T2 hyperintense round lesion at distal penile invading corpus spongiosum.

There was a 1 ∗ 2 cm erythematous plaque over the urethral meatus (Fig. 1), but no palpable lesion was observed in the penis or inguinal area on physical examination. All laboratory parameters were unremarkable, and the contrast-enhanced abdominopelvic CT scan was nonrevealing. Pelvic MRI shows 1.7 cm ∗ 1.7 cm T1intermidete (Fig. 2) and T2 hyperintense round lesion at distal penile invading corpus spongiosum but not corpus cavernous (Fig. 3). Flexible Urethrocystoscopy showed a nodular friable mass at the distal penile urethra with a flat erythematous lesion involving fossa navicular and meatus, and a cold cup biopsy showed squamous carcinoma in situ.

**Fig. 4:**
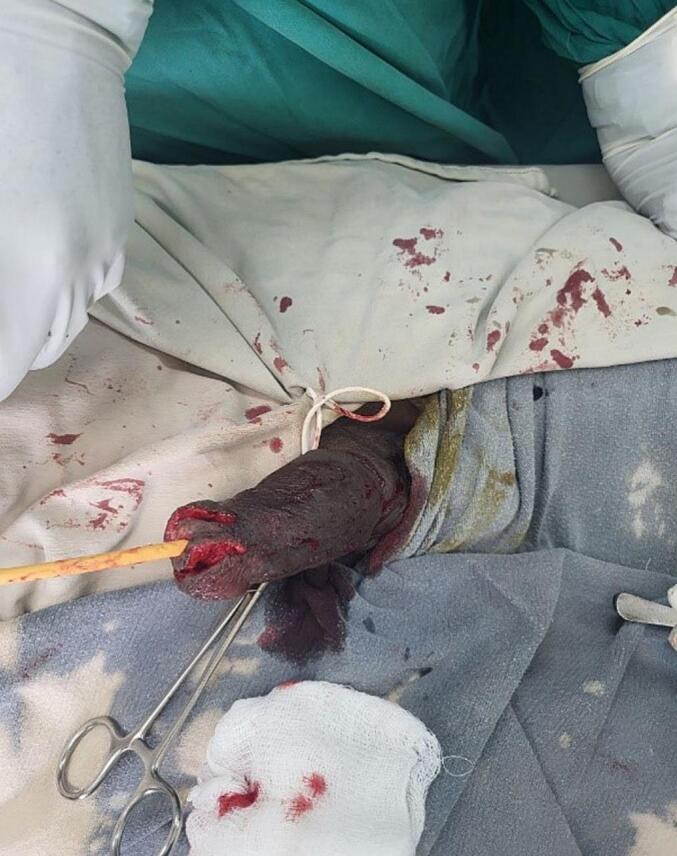
Intraoperative images showing partial glansectomy with 5 mm margin.

**Fig. 5:**
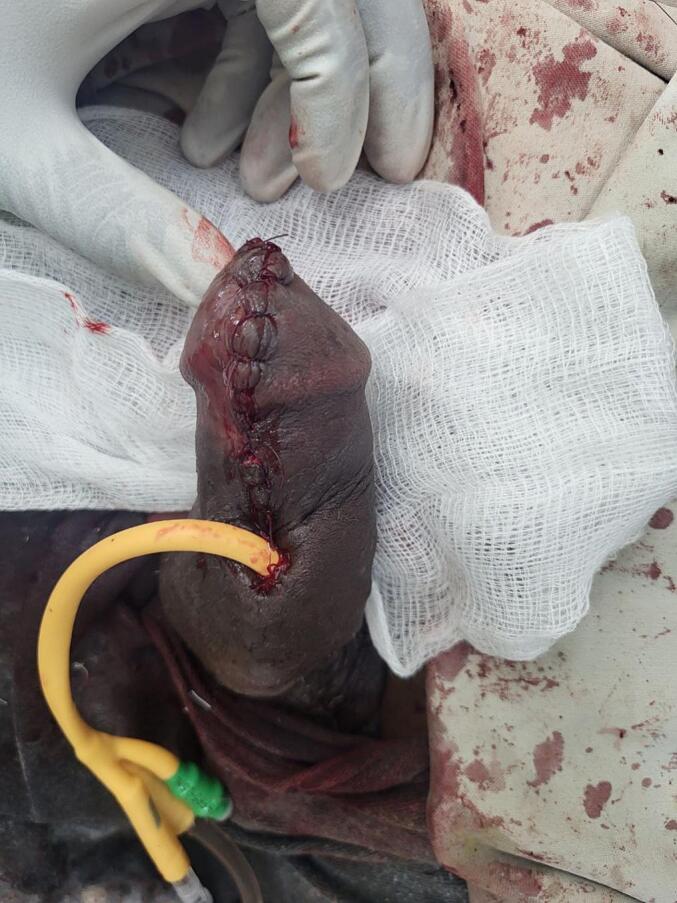
Intraoperative images showing partial anterior urethrectomy with proximal penile hypospadias meatus.

**Fig. 6:**
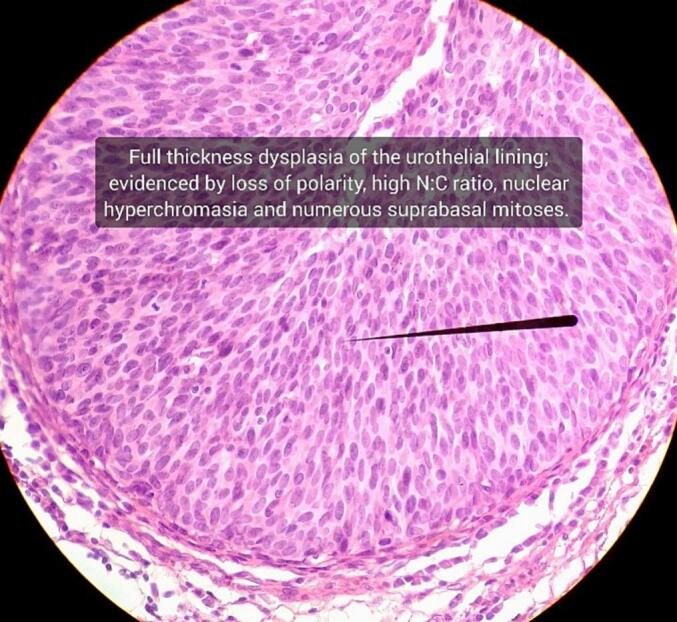
Histopathological sections show penile urethra predominantly lined by stratified columnar epithelium with areas of squamous metaplasia, full-thickness dysplasia.

**Fig. 7:**
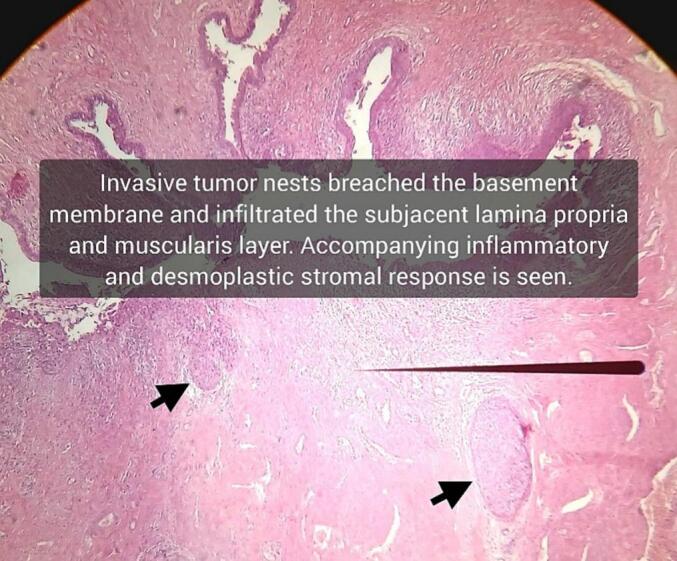
Focus on basement membrane integrity loss with pushing invasion into the subepithelial connective tissue.

**Fig. 8:**
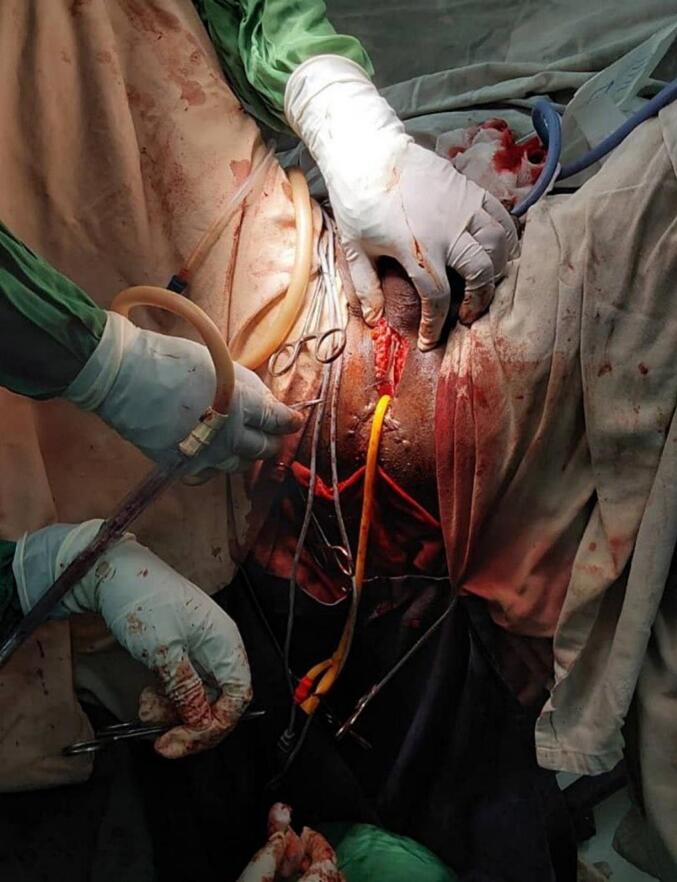
Intraoperative images total anterior urethrectomy with buccal mucosa graft augmented perineal urethrostomy.

**Fig. 9:**
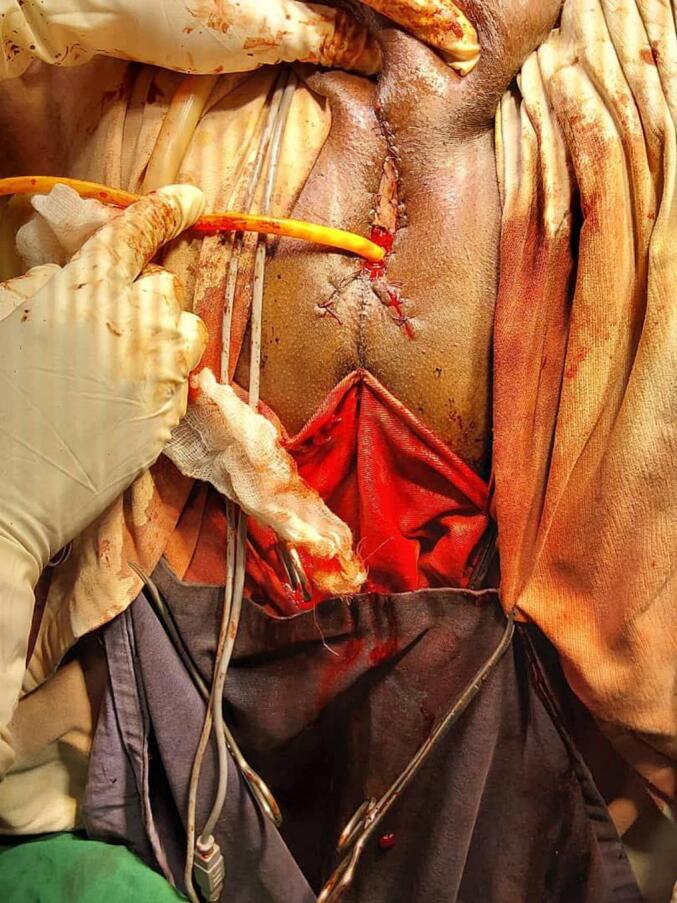
Intraoperative images of total anterior urethrectomy with buccal mucosa graft augmented perineal urethrostomy.

**Fig. 10:**
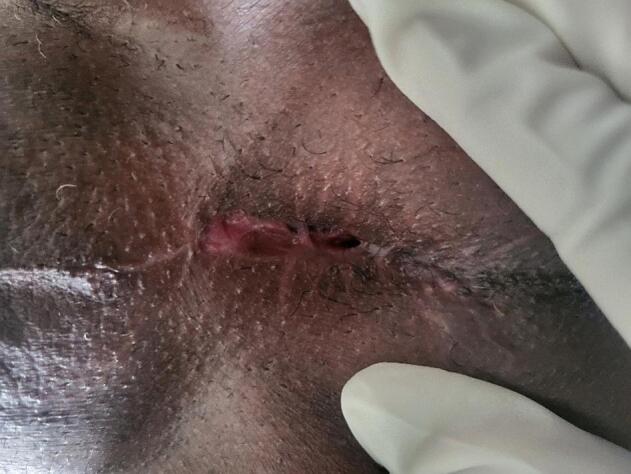
12-month postoperative image arrow head showing Functional perineal urethrostomy.

Since the biopsy showed squamous carcinoma in situ penile preservation was considered the patient subsequently underwent partial glansectomy and partial anterior urethrectomy with proximal penile meatoplasty (Fig. 4, Fig. 5). However, the pathology was well-differentiated squamous cell carcinoma invading the corpus spongiosum (Fig. 6, Fig. 7), but not cavernous, and the urethral margin was positive for the high-grade squamous intraepithelial lesion, frozen section was not taken due to service lack at time. Finally, a multi-disciplinary discussion was held, and due to limited radiotherapy sitting, on the 6th post-operative week surgical margin was managed by completion of anterior total urethrectomy with buccal mucosa graft-augmented perineal urethrostomy (Fig. 8, Fig. 9), after an intraoperative frozen section showed a negative margin. Currently, the patient is at 12 months post-operative follow-up (Fig. 10) with 6 monthly pelvic MRI and flexible cystoscopy and has a satisfactory quality of life (no urinary complaints and maintaining an erection).

## 3 Discussion

The presenting symptoms for PUC most commonly include obstructive voiding symptoms, hematuria or bloody urethral discharge, and a penile or perineal mass. Nearly all patients are symptomatic at the time of presentation [7]. Patients may also have a history of urethral stricture disease, sexually transmitted infections (specifically HPV-16), irritation from intermittent catheterization, and radiation exposure [1,6], which is consistent with our case. Owing to its nonspecific presentation, a high index of suspicion is required for the diagnosis of PUC.

As PUC is a rare cancer, there is sparse data available on its pathogenesis. It is believed that chronic inflammation and irritation of the urethra may play a role in the development of urethral cancer. Rapid turnover of urethral mucosal cells also predisposes individuals to the development of dysplasia and neoplasia [2,6]. However, the exact pathophysiological mechanisms involved in developing urethral cancer have not yet been elucidated.

The treatment for urethral carcinoma is not uniform [9]. Patients with proximal tumors often present with advanced disease and have poorer survival rates even with multimodal therapy involving surgery, chemotherapy, and radiotherapy [4,11]. However, patients with distal tumors tend to have better outcomes [6], as local surgical control can be achieved.

Urethral carcinoma has historically been managed by total penectomy with cystoprostatectomy for proximal tumors [12], and partial or radical penectomy for distal tumors with a 2 cm clear surgical excision margin similar to that of penile cancer [6,13]. However, studies have shown that resection margins of less than 5 mm, depending on the location and stage of the tumor, are also effective [14]. Treatments include creation of distal hypospadias with topical 5-fluorouracil (5-FU), distal urethrectomy with two-stage urethroplasty, glansectomy with skin graft reconstruction, and anterior urethrectomy with perineal urethrostomy [1,15]. Patients with positive margins were treated with additional surgery or adjuvant radiation, and none of the patients experienced local recurrence [16]. Squamous cell carcinoma in situ (CIS) of the peri-meatal glans, which can extend into the distal urethra, has been successfully treated with partial glansectomy and distal urethrectomy with urethral reconstruction or penile urethrectomy for anterior urethral cancer, resulting in no local recurrence [7,8,17,18]. Therefore, organ preservation surgery can be considered for carefully selected patients.

We followed a similar treatment modality for our patient, involving distal urethrectomy with partial glansectomy followed by completion anterior total urethrectomy with buccal mucosa graft-augmented perineal urethrostomy to surgically manage positive margins due to limited radiotherapy settings. Negative margins have been achieved, and no local recurrence has been identified to date. The outcome was oncologically satisfactory and the patient did not report any complaints of urinary symptoms or erectile dysfunction. Therefore, penis-preserving surgeries can be used safely, decreasing the psychological burden associated with total or partial penectomy, maintaining quality of life, and preserving functional outcomes.

As there are no established surveillance protocols for patients who have undergone organ-preserving treatment, it is recommended to individualize follow-up based on risk factors, involving more extensive follow-up with cytology, flexible cystoscopy, and cross-sectional imaging [2,12]. Our patient's follow-up will include flexible cystoscopy and cross-sectional imaging since the usefulness of urine cytology is yet to be determined.

We report the surgical management of a patient with distal urethral primary carcinoma presenting with a meatal ulcer, dysuria, bloody urethral discharge, and obstructive voiding symptoms. The condition was successfully managed with total anterior urethrectomy, partial glansectomy, and augmented perineal urethrostomy.

## 4 Conclusion

Primary urethral cancer is a rare urological malignancy that can be challenging to diagnose and treat. The clinical stage and anatomical location of the tumor are the most important clinical prognostic factors for urethral cancer in men. Current treatments favor penis-preserving surgery for distal disease, with a 5-year overall survival rate of approximately 50 %. Therefore, penis-preserving surgeries can be used safely, decreasing the psychological burden associated with total or partial penectomy, maintaining quality of life, and preserving functional outcomes.

## Author contribution

1. Alemu Bedado Hirpo (MD, Final year urology Resident) = Conceived, wrote, and reviewed the case report. Involved in the diagnosis, management, and follow-up of the patient and Operation on a patient under supervision.
2. Sena Sefera Akesa (MD, Assistant Professor of urology) = Conceived, wrote, and reviewed the case report. Supervised patient Operation.
3. Mensur Mohammed Ahmed (MD, Final year urology Resident) = Conceived, wrote, and reviewed the case report. Involved in the diagnosis, management, and follow-up of the patient and Operation on a patient under supervision.
4. Masresha Solomon Dino (MD, Assistant Professor of urology) = Conceived and reviewed the case report.
5. Wondweson Alemu Molla (MD, Pathology Resident) = Examined and reported the Histopathology. Reviewed the case report.
6. Mintesnot Yitagesu Kidane (MD, Urology Resident) = Reviewed the case report. Involved in the diagnosis, management, and follow-up of the patient.

## Consent

Written informed consent was obtained from a patient for publication and use of images. The written consent is available for review by the Editor-in-chief of this journal upon inquiry.

## Ethical approval

Ethical approval was deemed unnecessary by St. Paul's Hospital Millennium Medical College institutional review board as this is a single rare case faced during clinical practice and it doesn't involve experiments on humans and animal.

## Guarantor

Alemu Bedado Hirpo (Final year urology resident).

## Research registration number

Not applicable.

## Funding

No specific grant from funding organizations in the public, private, or nonprofit sectors was given to this manuscript.

## Conflict of interest statement

The authors declare no conflicts of interest.
